# Genome-wide screen for heavy alcohol consumption

**DOI:** 10.1186/1471-2156-4-S1-S106

**Published:** 2003-12-31

**Authors:** Diego F Wyszynski, Carolien I Panhuysen, Qianli Ma, Agustin G Yip, Marsha Wilcox, Porat Erlich, Lindsay A Farrer

**Affiliations:** 1 Genetics Program, Department of Medicine, Boston University School of Medicine, Boston, Massachusetts, 02118 USA

## Abstract

**Background:**

To find specific genes predisposing to heavy alcohol consumption (self-reported consumption of 24 grams or more of alcohol per day among men and 12 grams or more among women), we studied 330 families collected by the Framingham Heart Study made available to participants in the Genetic Analysis Workshop 13 (GAW13).

**Results:**

Parametric and nonparametric methods of linkage analysis were used. No significant evidence of linkage was found; however, weak signals were identified in several chromosomal regions, including 1p22, 4q12, 4q25, and 11q24, which are in the vicinity of those reported in other similar studies.

**Conclusion:**

Our study did not reveal significant evidence of linkage to heavy alcohol use; however, we found weak confirmation of studies carried out in other populations.

## Background

While heavy drinking is not necessarily indicative of alcohol abuse or alcoholism, individuals who binge drink are at a higher risk for alcohol-related disorders than others. "Heavy" (or "hazardous") drinking is a serious public health condition that has been defined in different ways. The Centers for Disease Control and Prevention describes it as "more than 14 drinks per week for men and more than 7 drinks per week for women". Using this definition, there are 8.7 percent of males and 6.7 percent of females who are heavy drinkers among current drinkers in the United States [1]. The U.S. Substance Abuse and Mental Health Services Administration defines "heavy alcohol use" as "drinking five or more drinks on the same occasion on each of 5 or more days in the past 30 days". According to this definition, more than 13% percent of young adults aged 18 to 25 were heavy alcohol users [2]. This percentage translates to approximately 4 million young adult heavy drinkers.

That alcoholism has a genetic component has been known for at least three decades and part of the Genetic Analysis Workshop 11 (GAW11) was dedicated to this phenotype [3]. Previous studies have shown evidence of linkage of alcoholism to markers on chromosomes 1, 2, 4, 7, and 11 [4-8]. To find specific genes predisposing to heavy alcohol consumption, we studied families collected by the Framingham Heart Study made available to participants in the Genetic Analysis Workshop 13.

## Methods

### Population

The Framingham Heart Study data set provided for the GAW13 included genotypes and longitudinal data for 330 families collected during 1948–1998 (original cohort) and between 1971–1999 (offspring cohort). "Heavy drinking" was defined as self-reported consumption of an average of more than 24 grams of alcohol per day during the year before the examination among men and an average of more than 12 grams per day among women. Data were available from 11 (out of 21) examinations in the original cohort and from all five examinations in the offspring cohort. Subjects who reported heavy drinking during the year previous to any one examination were classified as affected (original cohort *n *= 193; offspring cohort *n *= 578), whereas subjects who consistently reported no consumption of alcohol at any time in the year previous to all examinations were "unaffected" for the heavy drinking phenotype (original cohort *n *= 34; offspring cohort *n *= 53). The remaining family members, subjects who reported alcohol use in the year previous to at least one examination but who never consumed on average > 24 grams/day (men) or > 12 grams/day (women), were excluded from the analysis (original cohort *n *= 167; offspring cohort *n *= 677).

### Statistical analysis

Two-point parametric linkage analysis was performed by the VITESSE program [9]. Assuming the disease locus was at a given map position, we calculated the likelihood of the data using a range of different dominant and recessive transmission models with a fixed disease prevalence. The disease gene penetrance was assumed alternatively at 0.25, 0.50, 0.75, 0.85, and 0.99, while the phenocopy rate was tested at 0.01 and 0.001. The strategy of obtaining LOD scores using alternative models of inheritance has been tested successfully in several complex disorders [10,11].

Multipoint NPL (nonparametric linkage) analysis was performed using the S (pairs) option of GENEHUNTER-Plus, and maximized nonparametric LOD scores ("K&C LOD scores") were calculated under an exponential model with δ constrained between 0 and 2 [12].

Finally, two nonparametric affected sib-pair analyses were performed. Maximum-likelihood estimates of the proportions of sib pairs sharing 0, 1, or 2 alleles identical by descent (IBD) at marker loci were estimated with the routine SIB-MLS of the software GAS (v. 2.0) [13]. This nonparametric statistic is used to test for deviations of these proportions from the levels expected under the null hypothesis of no linkage. We also performed Haseman-Elston regressions as modified by Sham and Purcell [14] for all marker loci versus the trait using full and half-sib relative pairs as implemented in the software SIB-PAIR [15]. Asymptotic and empirical *p*-values were obtained. While this sib-pair linkage method was originally explained for a continuous trait, it is also applicable to binary traits [16].

## Results

There were 86 families with one heavy drinker, 73 with two, 59 with three, 38 with four, and 35 with five or more. One family had 16 heavy drinking members (26526). In all, four markers showed some evidence for linkage in parametric two-point LOD score analysis (Table 1); however, none reached a LOD score of 3.0.

Under the MLS affected sib-pair analyses, eight markers on six chromosomes yielded two-point MLS LOD scores ≥ 2.0 (Table 2) and the Haseman-Elston regression sib-pair analyses revealed six markers with asymptotic *p *< 0.01, of which four had empiric *p *< 0.01 after 201 iterations (Table 3). The multipoint NPL analysis did not reveal any suggestion of linkage (i.e., LOD score ≥ 2.0).

## Discussion

Assuming the recommendations of Lander and Kruglyak [17] that we accept, on average, one false positive in 20 genome scans (which corresponds to a nominal *p *= 0.000022 and LOD score of 3.63), our analyses failed to provide significant evidence of linkage to heavy alcohol use in the Framingham Heart Study data set. However, if we accept the suggestion of Rao and Gu [18] to relax the threshold enough to tolerate, on average, one false positive per 400-marker genome scan (which would correspond to *p *= 0.0023 and LOD score of 1.75), we identified several areas of potential interest, including 1p22, 11q24, and 12q. Our finding on chromosome 1 overlaps with that of Reich et al. [4], who reported linkage of alcoholism to 1p21-22. The dopamine receptor D2 (or DRD2) gene is located in 11q23. Because of its central role in the neuromodulation of appetitive behaviors, the DRD2 gene has been scrutinized as having a possible role in susceptibility to alcoholism, with mostly negative results [19,20]. We found a MLOD = 2.15 on 11q24, 15 cM downstream of DRD2. Its significance is uncertain. Two important alcohol-related enzymes are located close to chromosomal areas where we found weak LOD scores (~1.70): the aldehyde dehydrogenase 2 family (or ALDH2) is located on 12q24.2 and the alcohol dehydrogenase 1b, beta polypeptide (or ADH2) is in 4q22. We also had a "peak" (two-point MLOD = 1.72) at 4p13, where Long et al. [5] showed linkage in a Southwestern American Indian tribe.

Several reasons might explain our failure to find significant evidence of linkage to heavy alcohol use and the seemingly different results using alternative analytic methods. First, the phenotype we chose was extreme and yielded a low sample size, particularly of informative affected sib pairs. Second, individuals were classified as "affected" if they consumed, on average, high amounts of alcohol in the year previous to any examination. This might have been an inadequate choice, since it probably included a substantial number of heavy "social" drinkers, for whom a genetic susceptibility is unlikely. If the sample size had allowed it, we would have used the definition of the U.S. Substance Abuse and Mental Health Services Administration (see Background), which requires at least five episodes of heavy drinking in a 30-day period. Finally, the data on alcohol exposure was self-reported. We are unaware of reliability studies for alcohol consumption in this population. If misclassification bias exists, it might have reduced the statistical power and decreased the likelihood of finding significant results.

## Conclusion

Our study did not reveal significant evidence of linkage to heavy alcohol use; however, we found weak confirmation of studies carried-out in other populations.

## Figures and Tables

**Table 1:** Two-point parametric LOD scores for heavy alcohol use

**Chromosome**	**Marker**	**cM**	**LOD Score (θ)^A^**	**Model (penetrance)^B^**
1p22	GATA6A05	113.69	2.32 (0.20)	Recessive (0.85)
2p11	GATA71G04	99.41	2.56 (0.20)	Recessive (0.75)
4p12	GATA8A05	60.16	2.33 (0.00)	Dominant (0.25)
11q24	GATA64D03	123	2.15 (0.10)	Recessive (0.25)

^A^θ, recombination fraction. ^B^The disease gene penetrance was fixed alternatively at 0.25, 0.50, 0.75, 0.85, and 0.99. The phenocopy rate was tested at 0.01 and 0.001.

**Table 2:** Two-point maximum LOD score (MLS) for heavy alcohol use

**Chromosome**	**Marker**	**cM**	**MLS**
2q22	GATA26B04	149.89	2.88
4q12	GATA24H01	78.43	2.25
4q13	ATA2A03	93.48	2.49
6p21	GTAT1H06	53.81	2.11
14q21	GATA4B04	44.06	2.24
14q21	GGAA10C09	55.82	2.32
16p13	ATA41E04	11.46	2.19
19q13	mfd232	78.08	2.84

**Table 3:** Haseman-Elston regression sib-pair analysis for heavy alcohol use

**Chromosome**	**Marker**	**cM**	**Full Sibs**	**Half Sibs**	**t-Value**	**Asymptotic *p*-Value**	**Empiric *p*-value^A^**
4q25	ATA26B08	129.92	367	13	2.6	0.0047	0.0050
7q36	GATA4H10	161.21	74	0	2.7	0.0051	0.0149
7q36	Mfd265	181.97	387	17	2.6	0.0046	0.0100
11q25	ATA27C09	147.77	66	2	3.4	0.0008	0.0050
22q13	GATA21F03	28.57	383	19	3.0	0.0014	0.0050

^A^Based on 201 iterations.
